# Means of Applying Heat to Cholera Patients

**Published:** 1849-02

**Authors:** 


*Means of Applying Heat to Cholera Patients.*—Dr. William Robertson states that at the Cholera Hospital in Surgeon Square, Edinburgh, he has found the following means more efficacious for restoring warmth to cholera patients than the methods usually adopted for that purpose:

A sheet wrung out of warm water is applied, as hot as the patient can bear it, over his whole body, including and closely embracing the limbs, and leaving no part of the person but the head uncovered. Over the sheet several blankets are tightly wrapped, or 'packed,' after the fashion of the hydropaths, but without the slightest respect for their pathology, or wish to imitate what they can with justice claim as their exclusive practice. Between the folds of the blankets, vessels full of warm water are disposed at intervals. The patient is then placed in a position which enables him to vomit over the side of the bed, and is supplied with toast and water, hot or cold, *ad libitum.* The remedy is an ancient one, often revived in modern times, and is to be regarded merely as a simple and powerful hot-bath. Whether it acts by restoring the healthy functions of the skin, by preventing evaporation, or by conveying fluids into a system from which they have been previously drained away, may possibly admit of question. It certainly seems to me, when applied in the case of children suffering from the collapse of cholera, to be a most valuable and rapid mode of restoring the natural temperature. I have seen reaction established in a bad case within two hours after the application of the sheet. It is, however, generally necessary to continue the use of the remedy for six or eight hours. This practice seems less applicable to adults; extreme restlessness, jactitation, efforts to vomit and to procure drink, usually observed in such patients, render it quite impossible to continue the application of the sheet for more than a few minutes at a time, without more constant nursing than the utmost vigilance on the part of the medical attendants can, in an hospital, ensure. Strong patients commonly succeed, ere long, in disengaging their arms, and throwing the bedclothes off the upper part of the trunk, thereby exposing an extensive moist surface to evaporation, and totally defeating the object which we seek by the use of the sheet to attain."
—*Monthly Journal.*

